# PM2.5 Synergizes With *Pseudomonas aeruginosa* to Suppress Alveolar Macrophage Function in Mice Through the mTOR Pathway

**DOI:** 10.3389/fphar.2022.924242

**Published:** 2022-06-21

**Authors:** Jianlong Zhang, Chong Liu, Guangrong Zhao, Meng Li, Di Ma, Qingguo Meng, Wenli Tang, Qingrong Huang, Peimin Shi, Youzhi Li, Linlin Jiang, Xin Yu, Hongwei Zhu, Guozhong Chen, Xingxiao Zhang

**Affiliations:** ^1^ School of Life Sciences, Ludong University, Yantai, China; ^2^ Shandong Provincial Key Laboratory of Quality Safty Monitoring and Risk Assessment for Animal Products, Ji’nan, China; ^3^ Shandong Breeding Environmental Control Engineering Laboratory, Yantai, China; ^4^ School of Pharmacy, Yantai University, Yantai, China; ^5^ Yantai Key Laboratory of Animal Pathogenetic Microbiology and Immunology, Yantai, China; ^6^ Linyi Central Blood Station, Linyi, China

**Keywords:** PM2.5, *Pseudomonas aeruginosa*, alveolar macrophages, poultry houses, mTOR

## Abstract

High concentrations of PM2.5 in enclosed broiler houses cause respiratory disorders in humans and animals. *Pseudomonas aeruginosa* (*P. aeruginosa*) is an opportunistic pathogen that can induce severe respiratory disease in animals under stress or with abnormal immune functions. Alveolar macrophages are lung-resident immune cells that play important roles in lung host defence and immune balance. In this study, the mechanism by which PM2.5 synergizes with *P*. *aeruginosa* to damage alveolar macrophage function and induce inflammation was investigated. The results will provide a theoretical basis for improving the poultry breeding environment and preventing the recurrence of infection with *P*. *aeruginosa*. Alveolar macrophages were stimulated by PM2.5 collected in an enclosed broiler house and *P. aeruginosa*. Phagocytosis was determined by the neutral red test. The apoptosis rate and cytoskeleton changes were observed by flow cytometry assays and laser scanning confocal microscopy. Protein levels related to autophagy and the mTOR pathway were detected by Western blotting. The results indicated that PM2.5 in combination with *P. aeruginosa* could decrease phagocytosis, inhibit autophagy, increase apoptosis, and destroy the cytoskeleton in alveolar macrophages. In addition, alveolar macrophages had significantly increased expression of mTOR pathway-related proteins in response to the synergistic stimulation of PM2.5 and *P. aeruginosa*. The above results confirmed that PM2.5 in poultry houses synergized with *P. aeruginosa* to impede alveolar macrophage function and caused more severe respiratory system injuries through a process closely related to the activation of the mTOR signalling pathway.

## Introduction

Atmospheric particulate matter (PM) is the general term for all solid, liquid and aerosol substances in the atmosphere (Hunt et al., 2003). Microorganisms suspended as single cells in the air form suspended microbial aerosols by adhering to dry solid particles and liquid particles (Schlesinger and Cassee, 2003). With the development of the livestock and poultry breeding industry, the closed intensive breeding mode has gradually become mainstream. However, due to the high stocking density and poor air flow in these systems, a large number of microorganisms in the animal excreta and bedding in the houses and the hair and villi shed by the animals are likely to form PM with complex components. The pathogenic microbial components of such PM can enter the lungs through the respiratory tract and cause inflammation, greatly endangering the health of livestock and breeding personnel (Lawniczek-Walczyk et al., 2013). The particle size determines the specific surface area of the harmful substances absorbed by PM, as well as the depth to which PM enters the respiratory tract. Particulates with a diameter ≤2.5 μm are called PM2.5. These particles have a larger specific surface area and are more likely than smaller particulates to combine with organic compounds, heavy metals, and microbial components in the air. PM2.5 can enter the deep part of the respiratory tract and are not easily excreted from the body. They are deposited in the bronchi, bronchioles, and alveoli of the lungs, and some PM2.5 components can even cross the pulmonary interstitium and enter the blood, seriously affecting the health of humans and animals (Franck et al., 2011). The damage caused by PM2.5 to the respiratory system includes respiratory infections, lung function decline, bronchitis, chronic obstructive pulmonary disease, and even lung cancer (Hsieh et al., 2008). In addition, PM2.5 can damage the immune system, cardiovascular system, and reproductive system (Schwartz, 2004).

*Pseudomonas aeruginosa* (*P*. *aeruginosa*) is unlikely to cause disease in healthy individuals but tends to cause serious infections in patients with compromised or defective immune functions. *P. aeruginosa* is a very important pathogen that causes human pulmonary infection. It often causes respiratory tract infections, cystic fibrosis, diffuse panbronchiolitis, pneumonia, and many other diseases (Cachia and Hodges, 2003). *P. aeruginosa* can also cause disease in poultry, leading to pulmonary infection, septicaemia, and even death, with serious losses to the breeding industry (Walker et al., 2002).

Alveolar macrophages are derived from bone marrow and are mainly distributed on the epithelial surface of the bronchial tract and in the alveoli. They are the first immune defence cells in contact with foreign bodies or microorganisms. These macrophages have strong phagocytic ability and can nonspecifically phagocytose a variety of pathogens, playing an important role in host defence and immune balance in the lungs. Under normal circumstances, alveolar macrophages can protect against PM or microorganisms entering the alveolar space through phagocytosis and prevent lung tissue damage through secretion and immune regulation (Byrne et al., 2015).

Our previous studies have shown that macrophages significantly increase the expression levels of inflammatory cytokines, such as tumour necrosis factor (TNF)-α, interleukin (IL)-6 and IL-8, as well as nuclear factor-κB (NF-κB) pathway-related proteins under the synergistic stimulation of PM2.5 and *P. aeruginosa* in poultry houses (Li et al., 2020). These findings indicate that high concentrations of PM2.5 can damage the respiratory system and cause secondary infections of *P. aeruginosa*, resulting in more severe respiratory system damage. This process is closely related to the activation of the NF-κB pathway. It has been reported that there is crosstalk between the NF-κB and mammalian target of rapamycin (mTOR) pathways, and the mTOR pathway is closely related to macrophage apoptosis, autophagy, and phagocytosis (Kim et al., 2007; Zoncu et al., 2011). In this study, we used PM2.5 in poultry houses together with *P. aeruginosa* to stimulate alveolar macrophages to establish a cell model and detect apoptosis, autophagy, and phagocytosis, as well as the expression of mTOR pathway-related proteins, to elucidate the molecular mechanisms of the mTOR signalling pathway in response to damage by alveolar macrophages and the induction of respiratory tract inflammation by PM2.5 and *P. aeruginosa*. This study helps clarify how PM2.5 and *P. aeruginosa* damage the respiratory system of humans and animals and induce an inflammatory response, and the results provide a theoretical basis for the prevention and treatment of secondary infection by *P. aeruginosa*.

## Experimental Materials and Methods

### Ethical Statement

All animal experimental protocols were performed following requirements and management guidelines that were reviewed and approved by the Animal Ethics and Experimental Committee of Ludong University (Permit Number: LDU-IACUC2018007). Mice were injected with anaesthetics (Zoletil 50, Virbac, France) according to the instructions before sacrifice. No endangered animals were involved in the experiment, and the pain of the animals was minimized as much as possible during the operation.

### PM2.5 Sampling and Processing

The sampling site was a chicken house in Muping, Yantai, Shandong Province, China (37°25′3.78″N, 121°40′33.29″E). The sampling period was from 24 July 2019 to 24 August 2019. During the experiment, an air PM sampler (ZR-3920, China) was used for sampling at a flow rate of 100 L/min. The sampler was placed 1 m above the ground in the centre of the poultry house for sampling, and the sampling duration was 99 h. PM2.5 was collected with 9 cm × 9 cm waterproof glass fibre filter membranes, and the filter membranes were sterilized by dry heat before sampling (Zhang et al., 2019). To better evaluate the effect of the microorganisms carried by PM2.5 on alveolar macrophages, we divided the collected filter membranes into two groups, one of which was treated with heat inactivation (PM2.5^—^) and the other without heat inactivation (PM2.5). During the experiment, PM2.5 was dissolved and diluted to the desired concentration with sterile pyrogen-free phosphate-buffered saline (PBS) as needed.

### Animals

Eight-week-old SPF male C57BL/6 mice with a body weight of 24 ± 2 g were purchased from Jinan Pengyue Experimental Animal Co., Ltd., and were used for the experiments. The temperature of the breeding environment was 23–25°C, the humidity was 50 ± 10%, and the photoperiod was 12/12 h. During the feeding period, the mice had *ad libitum* access to movement, food, and water.

### Bacterial Culture

The *P. aeruginosa* PAO1 strain was maintained in our laboratory. After activation, the PAO1 strain was cultured and characterized by streaking on CN agar identification plates and incubating at 37°C for 24 h. Then, single colonies were gathered, inoculated into LB liquid medium, and incubated with shaking at 37°C and 170 rpm to expand the culture. During the experiment, the concentration of the bacterial culture was diluted to the required concentration with sterile pyrogen-free PBS.

### Isolation and Culture of Alveolar Macrophages

After the mouse was anaesthetized and disinfected, the thoracic cavity of the mouse was opened, and its airway was exposed. Prechilled alveolar lavage buffer (1 mM EDTA, Ca^2+^- and Mg^2+^-free PBS solution) was used for flushing, and the alveolar lavage fluid was collected. The collected alveolar lavage fluid was centrifuged at 250 g for 10 min at 4°C. The collected alveolar macrophages were resuspended in Dulbecco’s modified Eagle’s medium (DMEM) (containing 10% foetal bovine serum (FBS), 20% L-929 cell culture medium, 1 mM sodium pyruvate, 10 mM 4-(2-hydroxyethyl)-1-piperazineethanesulfonic acid (HEPES), and 1 × penicillin/streptomycin) and were seeded into cell culture flasks (Nayak et al., 2018).

Subsequent experiments were performed using isolated alveolar macrophages. Six groups were established in the experiment: a normal control group (PBS), a PM2.5 non-inactivated group (PM2.5), a PM2.5 inactivated group (PM2.5^—^), a PAO1 group (PA), a PM2.5 non-heat-inactivated + PA group (PM2.5 + PA), and a PM2.5 heat-inactivated + PA group (PM2.5^—^ + PA). In each treatment group, the final concentration of PM2.5 was 100 μg/ml, and the multiplicity of infection (MOI) of *P. aeruginosa* was 10:1 (the number of bacteria: the number of cells). The control group was mixed with the corresponding volume of PBS.

### Neutral Red Phagocytosis Assay

The cell concentration was adjusted to 3 × 10^5^ cells/ml, and the cells were seeded into a 96-well plate at 100 μl per well. After incubation at 37°C and 5% CO_2_ for 24 h, follow-up experiments were performed. Each group was added to a corresponding volume of samples and stimulated for 12 h at 37°C with 5% CO_2_. The supernatant in each well was discarded, and the cells were washed twice with PBS before the detection of the ability of cells to phagocytose neutral red (Cui et al., 2021). The experimental process was performed in strict accordance with the instructions of the neutral red detection kit (Beyotime).

### Detection of Apoptosis

The alveolar macrophages were diluted to 2 × 10^5^ cells/ml, and the cells were seeded into 24-well plates at 500 μl per well. After incubating at 37°C and 5% CO_2_ for 24 h, follow-up experiments were performed. Each group was mixed with a corresponding volume of samples and stimulated for 12 h before subsequent experiments were conducted. The cells were washed twice with PBS and trypsinized, after which the cells were harvested. The experiment was performed according to the instructions of the Annexin V-FITC/PI kit (Beyotime) to detect cell apoptosis by flow cytometry (Engelbrecht et al., 2004).

### Cytoskeletal Observations

The cells were seeded into coverslip-containing six-well plates at 3 × 10^5^ cells/well and cultured for 24 h at 37°C and 5% CO_2_ before subsequent experiments. Each group was stimulated with a corresponding volume of sample for 12 h before subsequent experiments. The cells were first fixed on ice for 15 min using a PBS solution containing 3.75% formaldehyde. They were washed and then permeabilized with 0.5% Triton X-100 in PBS for 10 min at room temperature. The cells were rinsed again, and then 200 μl of phalloidin working solution was added and incubated at room temperature for 20 min for staining. The cells were rinsed, stained with 200 μl of 4′,6-diamidino-2-phenylindole (DAPI) staining solution, and observed under a laser confocal microscope (Wang et al., 2019).

### Western Blotting

The cells were seeded into coverslip-containing six-well plates at 3 × 10^5^ cells/well and cultured for 24 h at 37°C and 5% CO_2_ before subsequent experiments. Each group was stimulated with the corresponding volume of sample for 12 h before the follow-up experiments were performed. Cells were collected with a cell scraper and lysed by adding RIPA lysis buffer (Solarbio, China) containing protease inhibitors and phenylmethylsulfonyl fluoride (PMSF) for 30 min. Cellular proteins were extracted by centrifugation at 12,000 rpm for 15 min. After the protein concentration was determined with the bicinchoninic acid (BCA) method, equal amounts of protein in each group were loaded onto a sodium dodecyl sulfate–polyacrylamide gel electrophoresis (SDS–PAGE) gel and transferred to a polyvinylidene fluoride (PVDF) membrane. The membrane was blocked for 2 h with 5% fat-free milk. Rabbit anti-mouse LC3II antibody (Cell Signalling Technology, United States, 1:1,000), rabbit anti-mouse p62 antibody (Cell Signalling Technology, United States, 1:1,000), rabbit anti-mouse mTOR antibody (Cell Signalling Technology, United States, 1:1,000), rabbit anti-mouse p-mTOR antibody (Cell Signalling Technology, United States, 1:1,000), rabbit anti-mouse Akt antibody (Cell Signalling Technology, United States, 1:1,000), rabbit anti-mouse p-Akt antibody (Cell Signalling Technology, United States, 1:1,000), and β-actin antibody (Proteintech, United States, 1:5,000) were incubated for 2 h at room temperature. Goat anti-rabbit IgG Dylight 800 antibody (Cell Signalling Technology, United States, 1:30,000) was incubated for 1 h at room temperature in the dark. Protein expression was detected using a fluorescence imager (LI-COR, United States), and relative grey values were calculated (Zhang et al., 2020).

### Data Analysis

Statistical analysis was performed using SPSS V17.0 (SPSS Inc., Chicago, IL, United States), and figures were generated using GraphPad Prism version 7.0 (GraphPad Software Inc., San Diego, CA, United States). The data are presented as the means ± SDs of at least three independent experiments. The statistical significance of differences between groups was determined by the unpaired *t* test or one-way analysis of variance (ANOVA). Differences were considered statistically significant for *p* values <0.05.

## Experimental Results

### PM2.5 and *Pseudomonas aeruginosa* Exposure Decreases Phagocytosis of Cells

**FIGURE 1:**
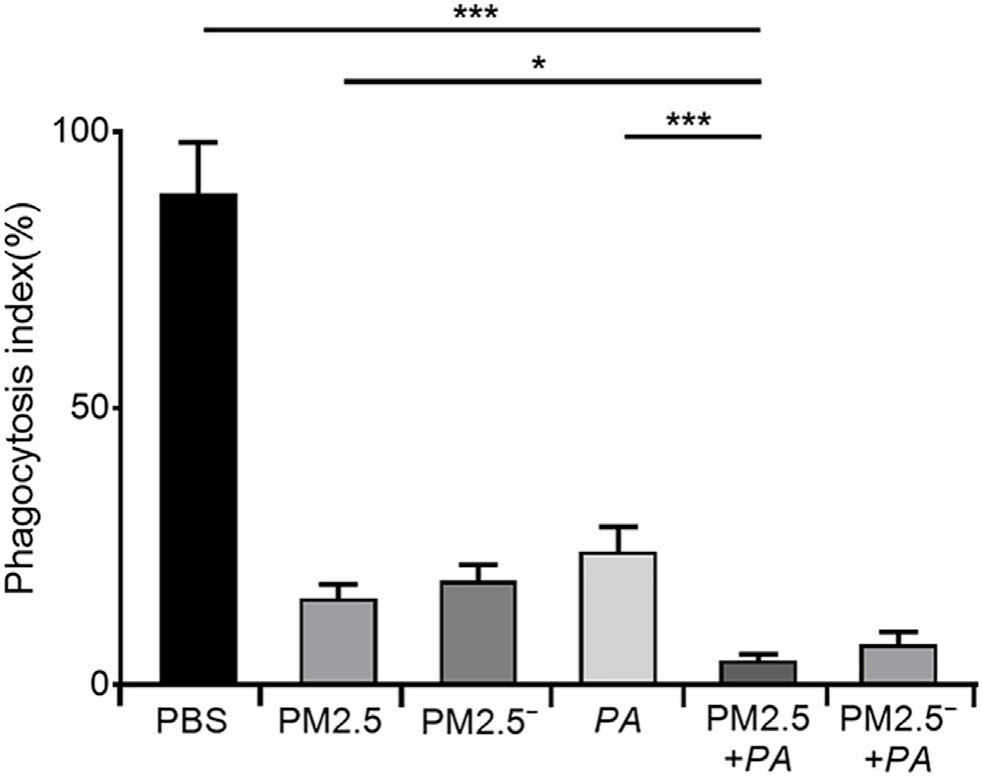
Costimulation by PM2.5 and *P. aeruginosa* can reduce the phagocytic ability of mouse alveolar macrophages. In the experimental groups, the final concentrations of the PM2.5 and PM2.5^—^ samples were 100 μg/ml, and the multiplicity of infection (MOI) between *P. aeruginosa* and the cells was 10:1 (number of bacteria:number of cells). Each group was stimulated for 12 h with the corresponding sample, and the control group was treated with an equal volume of PBS. The experiment was repeated three times. PBS, phosphate-buffered saline; PM2.5, particulate matter less than 2.5 μm in diameter; *P. aeruginosa*, *Pseudomonas aeruginosa*. The data are presented as the means ± SD. One-way ANOVA in SPSS. *p < 0.05, **p < 0.01, ***p < 0.001.

To more accurately analyse the effect of PM2.5 and *P. aeruginosa* on the phagocytic ability of alveolar macrophages, we performed a neutral red phagocytosis assay. The results are shown in Figure 1. Compared with the unstimulated normal cells, the phagocytic ability of cells in other groups was lower (*p* < 0.05), and stimulating the cells with PM2.5 and *P. aeruginosa* simultaneously had the most significant effect on the phagocytic ability of cells (*p* < 0.001).

### PM2.5 and *Pseudomonas aeruginosa* Mediate Macrophage Apoptosis

**FIGURE 2:**
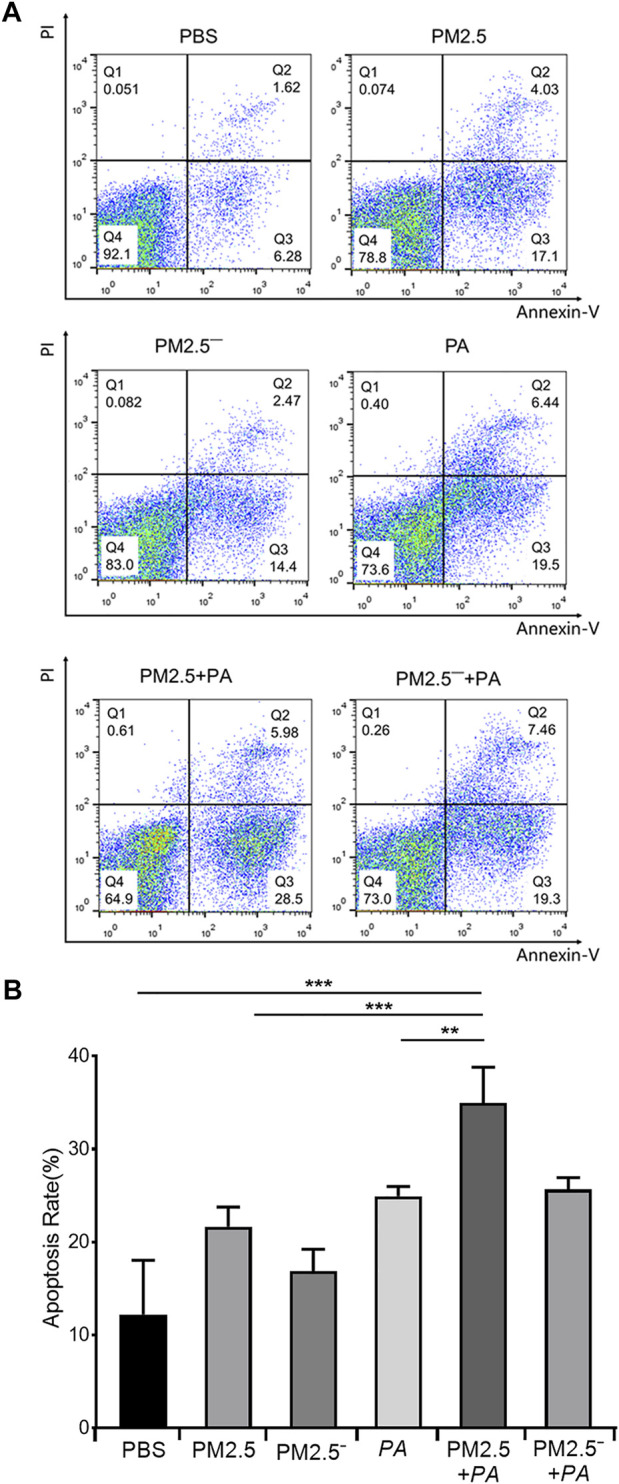
Costimulation by PM2.5 and *P. aeruginosa* can aggravate the apoptosis of mouse alveolar macrophages. **(A)** Flow cytometry was used to detect apoptosis in each group after 12 h of stimulation with the samples. **(B)** The proportion of apoptotic cells in each group. In the experimental groups, the final concentration of the PM2.5 and PM2.5^—^ samples was 100 μg/ml, and the multiplicity of infection (MOI) between *P. aeruginosa* and the cells was 10:1 (number of bacteria:number of cells). Each group was stimulated for 12 h with the corresponding sample, and the control group was treated with an equal volume of PBS. The experiment was repeated three times. PBS, phosphate-buffered saline; PM2.5, particulate matter less than 2.5 μm in diameter; *P*. *aeruginosa*, *Pseudomonas aeruginosa*. The data are presented as the means ± SD. One-way ANOVA in SPSS. **p < 0.01; ***p < 0.001.

In the detection of the effects of PM2.5 and *P. aeruginosa* on apoptosis using flow cytometry, it was found that the apoptosis rates of the other groups were higher than that of normal unstimulated cells (7.90%), as shown in Figure 2. The apoptosis rate of the PM2.5 + PA group was 34.48%, which was significantly higher than those of the PM2.5 group and the *P. aeruginosa* group (21.13% and 23.94%, respectively, *p* < 0.01). This finding indicated that the combined action of PM2.5 and *P. aeruginosa* was able to significantly promote apoptosis of alveolar macrophages.

### Effect of PM2.5 and *Pseudomonas aeruginosa* on the Cytoskeleton of Macrophages

**FIGURE 3:**
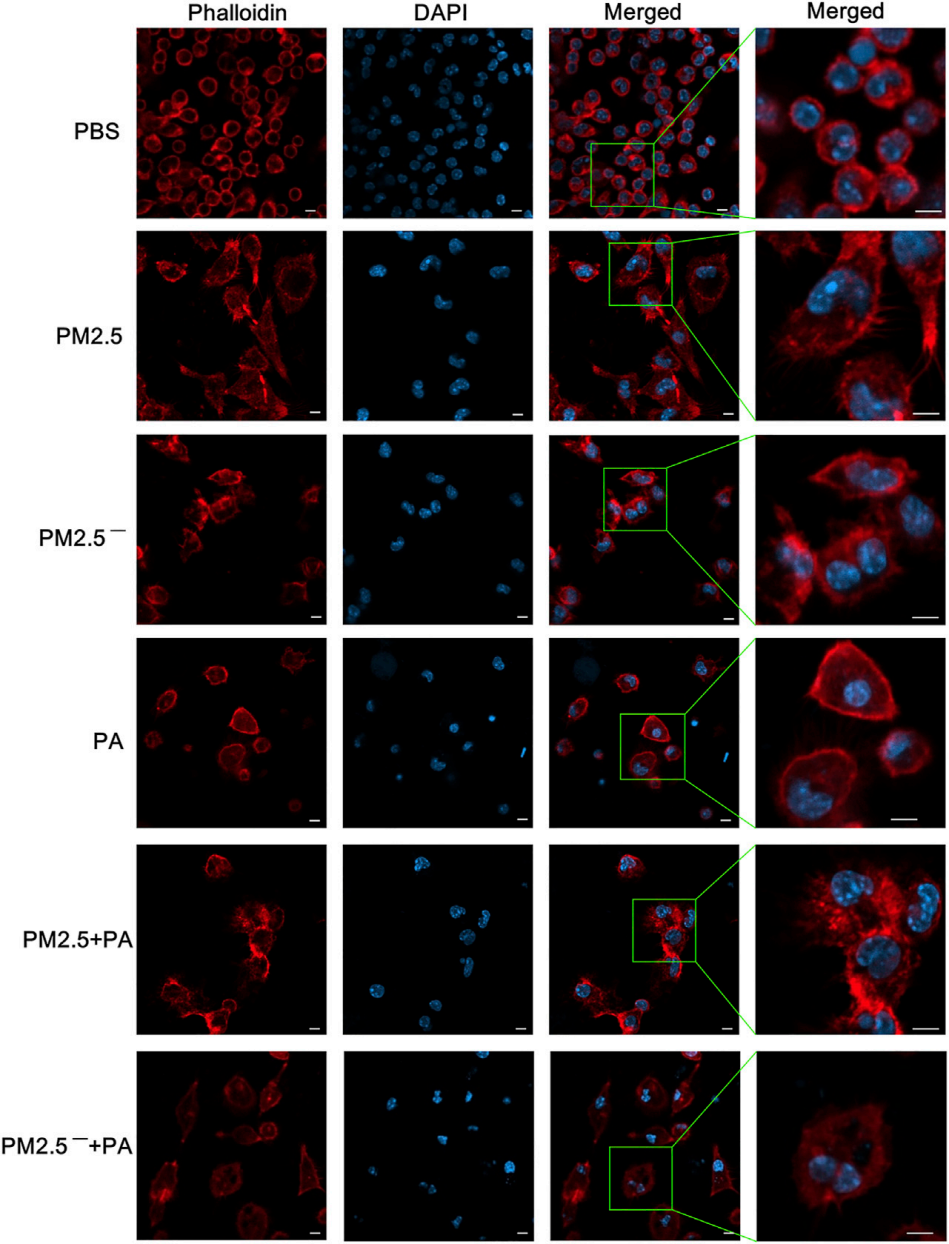
The combined action of PM2.5 and *P. aeruginosa* can cause changes to the cytoskeleton. After phalloidin and DAPI staining, laser confocal microscopy was used to observe the cytoskeletal changes in cells in each group stimulated by samples for 12 h. In the experimental groups, the final concentrations of the PM2.5 and PM2.5^—^ samples were 100 μg/ml, and the multiplicity of infection (MOI) between *P. aeruginosa* and the cells was 10:1 (number of bacteria:number of cells). An equal volume of PBS was added to the control group. Red, phalloidin; blue, DAPI; scale bar, 5 μm. The experiment was repeated three times. PBS, phosphate-buffered saline; PM2.5, particulate matter less than 2.5 μm in diameter; *P*. *aeruginosa*, *Pseudomonas aeruginosa*.

The results of laser confocal microscopy (Figure 3) showed that the cells and nuclei of the normal group were morphologically normal, showing a round or nearly round shape with a smooth cell membrane surface. However, in the experimental group, the cell status was abnormal, mainly manifested as polygonal cell morphology, an uneven cell membrane surface, and the appearance of more pseudopodia. Additionally, the nuclear morphology changed, the cytoplasm expanded, and the nuclei of the PM2.5 group showed irregular morphology. In the experimental groups, the changes in cells in the PM2.5 + PA group were the most substantial, and the cells in this group no longer had the morphology of normal cells but had a disordered microfilament structure. This outcome shows that PM2.5 and *P. aeruginosa* can destroy the skeletal structure of alveolar macrophages, and the effect is greatest when the two act synergistically.

### PM2.5 and *Pseudomonas aeruginosa* Exposure Suppresses Autophagy in Macrophages

**FIGURE 4:**
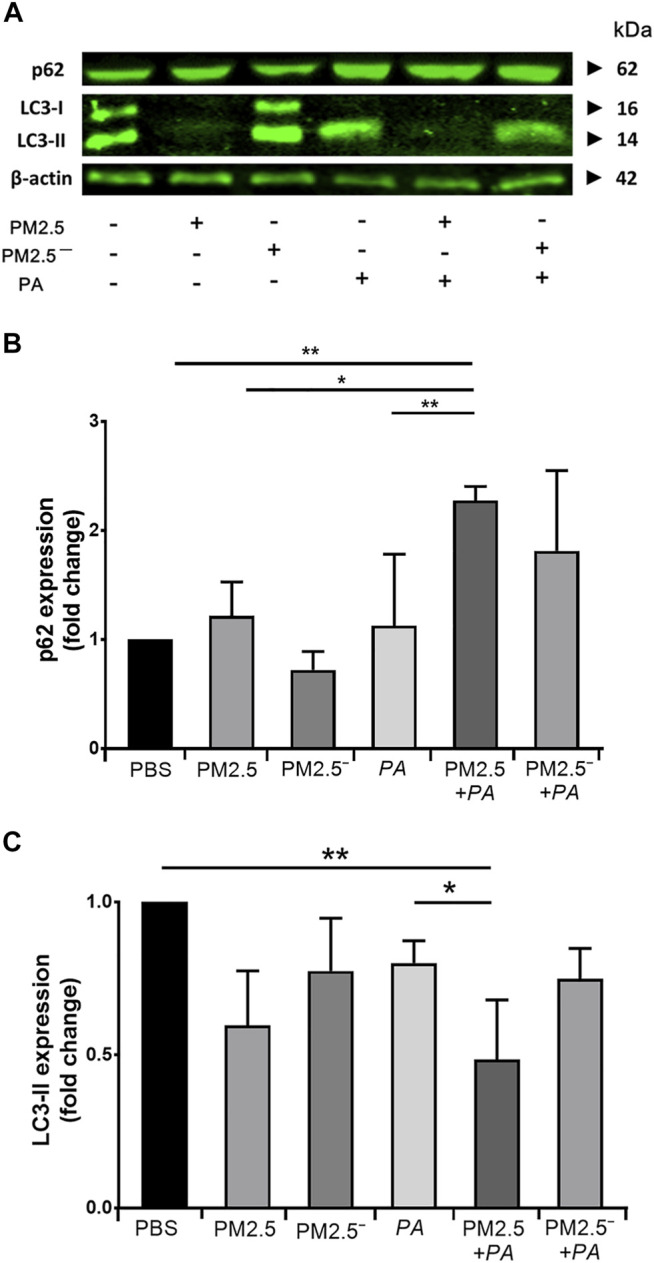
Costimulation by PM2.5 and *P. aeruginosa* can promote autophagy. **(A)** The expression levels of p62 and LC3 proteins in the cells were detected by Western blotting. **(B,C)** The expression levels of p62 and LC3 in the cells of each group. The experiment was repeated three times. PBS, phosphate-buffered saline; PM2.5, particulate matter less than 2.5 μm in diameter; *P*. *aeruginosa*, *Pseudomonas aeruginosa*. The data are presented as the means ± SD. One-way ANOVA in SPSS. *p < 0.05, **p < 0.01.

After alveolar macrophages were treated, the protein expression levels of LC3 and p62 in the cells of each group were detected by Western blotting. The expression of p62 protein is shown in Figures 4A,B. The expression of p62 protein was upregulated in all groups except for the PM2.5^—^ group. Among them, the upregulation of p62 was the most significant under the synergistic stimulation of PM2.5 and *P. aeruginosa* (*p* < 0.05). In addition, all of the groups of samples had reduced expression of LC3-II to varying degrees. Compared with the blank control group, concerted stimulation with PM2.5 and *P. aeruginosa* significantly downregulated the expression of LC3-II (*p* < 0.05). The expression of p62 and LC3-II can reflect the level of autophagy to a certain extent (Du et al., 2019). This finding suggests that combined stimulation by PM2.5 and *P. aeruginosa* can significantly inhibit autophagy levels in alveolar macrophages.

### Importance of Akt/mTOR in the Response to PM2.5 and *Pseudomonas aeruginosa*

**FIGURE 5:**
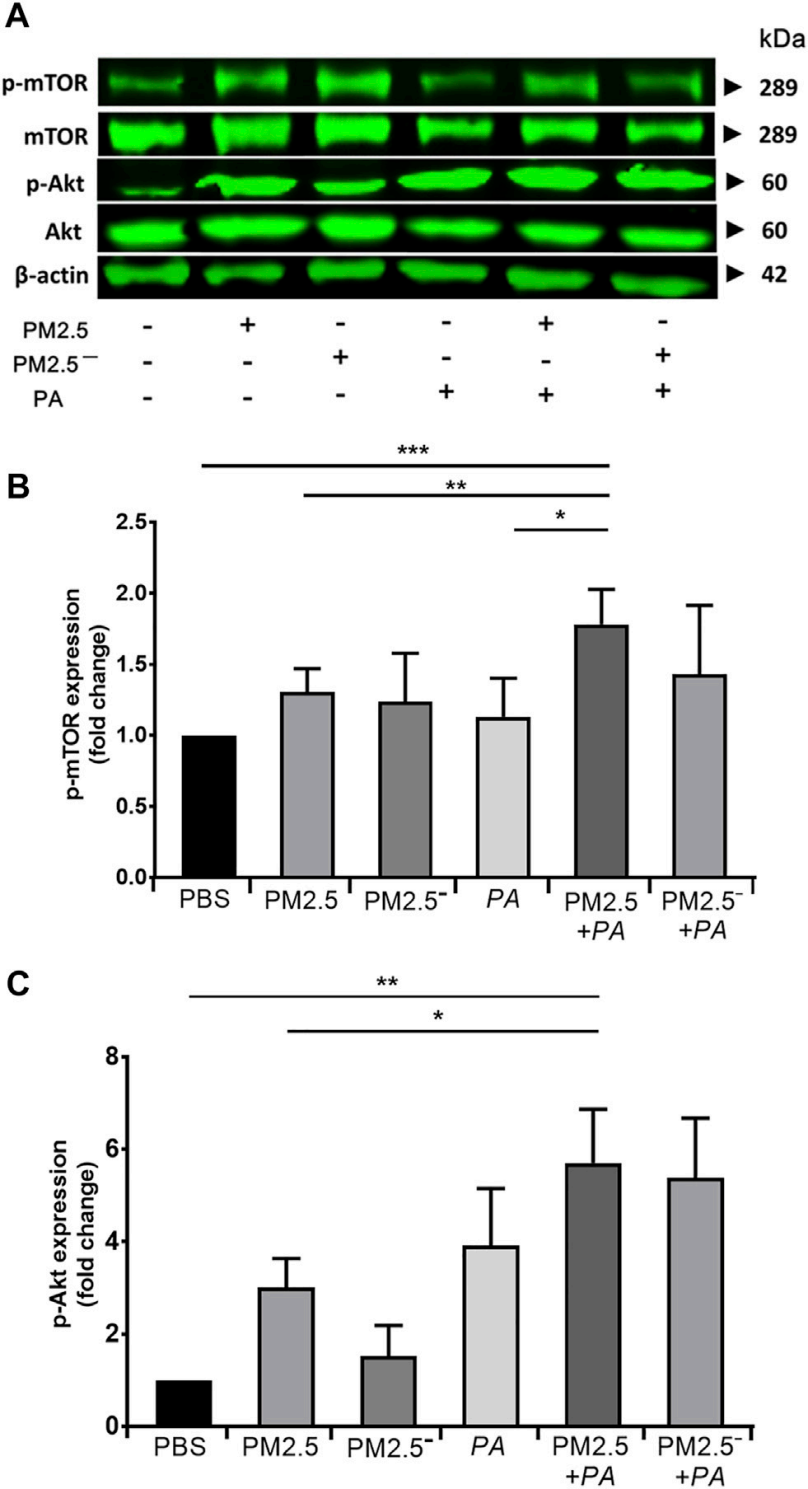
Costimulation by PM2.5 and *P. aeruginosa* can affect the Akt/mTOR signalling pathway. **(A)** The protein expression levels of Akt, p-Akt, mTOR, and p-mTOR in cells were detected by Western blotting. **(B)** The protein expression level of phospho-mTOR in cells of each group. **(C)** The protein expression level of phospho-Akt in cells of each group. The experiment was repeated three times. PBS, phosphate-buffered saline; PM2.5, particulate matter less than 2.5 μm in diameter; *P*. *aeruginosa*, *Pseudomonas aeruginosa*. The data are presented as the means ± SD. One-way ANOVA in SPSS. *p < 0.05, **p < 0.01, ***p < 0.001.

Real-time PCR and Western blotting were used to detect the expression of Akt/mTOR signalling pathway-related genes and proteins in alveolar macrophages, respectively. The results of Western blotting (Figure 5) showed that the expression of Akt, p-Akt, mTOR, and p-mTOR was enhanced to a certain extent in all of the groups compared with that in the blank control group, and the protein expression was significantly increased when PM2.5 and *P. aeruginosa* acted together (*p* < 0.01). This outcome suggests that compared with stimulation by PM2.5 or *P. aeruginosa* alone, the combined action of PM2.5 and *P. aeruginosa* activated the Akt/mTOR signalling pathway to a greater extent.

## Discussion

In recent years, many studies have shown that the increasing incidence and mortality of lung diseases are closely related to the increase in atmospheric PM2.5 concentrations (Annesi-Maesano et al., 2007). Alveolar macrophages are free immune cells in the lung. They are generally derived from bone marrow and are mainly distributed on the surface of the respiratory tract and alveoli. They can eliminate foreign bacteria through phagocytosis and digestion. Therefore, they are referred to as the first line of defence against the invasion of foreign microorganisms in the lungs and airways. The lungs mainly rely on alveolar macrophages to phagocytose foreign PM or microorganisms and then break them down and remove them through lysosomes (Allard et al., 2018). Many studies have shown that PM2.5 can significantly reduce the phagocytic ability of alveolar macrophages *in vivo* and *in vitro* experiments, and as the concentration of PM increases, the phagocytic rate and phagocytic index of alveolar macrophages significantly decrease (Miyata and van Eeden, 2011). Our experimental results also confirmed that mouse alveolar macrophages had weaker phagocytosis and lower resistance to pathogenic bacteria after inhalation of PM2.5. Therefore, *P. aeruginosa* had the opportunity to adhere to and colonize the surface of the respiratory tract, which could cause greater damage to the phagocytic function of alveolar macrophages.

Apoptosis is active cell death under the control of genes, and it is an inherent programmed biological phenomenon that widely occurs in many physiological and pathological processes of cells. Apoptosis can be induced by physiological stimulation signals or by external environmental factors (Elmore, 2007). Apoptosis maintains the dynamic balance of cell proliferation and cell death in living organisms, thereby maintaining a constant number of cells and normal physiological function in the organism. Abnormal regulation of apoptosis (insufficient activation of apoptosis, inappropriate activation, or inhibition of apoptosis) leads to the occurrence of various diseases (Fleisher, 1997). Many studies have shown that PM2.5-induced pulmonary cell apoptosis is an effective host defence mechanism that can effectively reduce the damage by PM2.5 to the host. Long-term exposure to an environment with a PM2.5 level greater than the hazard threshold value can easily disrupt the apoptotic balance of the respiratory system and induce lung injury (Xia et al., 2017). In the PM2.5-induced acute lung injury model, PM2.5 can stimulate the generation of reactive oxygen species, which activate the phosphorylation site Thr845 of ASK1 and thus activate the p38 and JNK signalling pathways and cause apoptosis of airway epithelial cells, eventually leading to acute lung injury (Ni et al., 2015). The results of apoptosis experiments showed that both PM2.5 and *P. aeruginosa* could promote the apoptosis of mouse alveolar cells, and concerted stimulation by PM2.5 and *P. aeruginosa* could aggravate the apoptosis of mouse alveolar macrophages. These outcomes indicate that PM2.5 can induce apoptosis of alveolar macrophages, disrupt the apoptotic balance of macrophages, and damage the defence function of the lungs. Pathogenic bacteria, such as *P. aeruginosa,* are more likely to colonize the lungs and further induce apoptosis of alveolar macrophages. Excessive apoptosis can lead to severe inflammatory responses and cause lung injury (Wang et al., 2005).

The cytoskeleton is a complex network-like structural system that runs through the interior of eukaryotic cells and plays an important role in phagocytosis by macrophages. The cytoskeleton can not only maintain cell morphology but can also participate in cell division and motility, intracellular substance transport, phagocytosis, and other functions, and it has important regulatory functions in all aspects of signal transduction. The cytoskeleton is composed of microtubules, intermediate filaments, and microfilaments, and the main component of microfilaments is actin (Moller et al., 2005). The correct rearrangement of cytoskeletal actin is necessary for phagocytosis by macrophages (May and Machesky, 2001). Macrophage membranes undergo morphological changes and extend pseudopodia, lamellipodia, or phagocytic cups under the continuous polymerization and depolymerization of cytoskeletal actin to engulf pathogens and form phagolysosomes, thereby clearing pathogens (Allen and Aderem, 1996; Egami et al., 2017). In this study, we observed cytoskeletal changes in alveolar macrophages using laser confocal microscopy. The experimental results were consistent with the findings of phagocytosis experiments. Combined stimulation by PM2.5 and *P. aeruginosa* caused the most severe damage to the cytoskeleton of alveolar macrophages and significantly inhibited phagocytosis by alveolar macrophages. This outcome also shows that high concentrations of PM2.5 could lead to impaired rearrangement of the cytoskeleton of mouse alveolar macrophages and that the delayed phagocytosis of *P. aeruginosa* further affects the cytoskeleton of alveolar macrophages, causing significantly decreased phagocytic ability.

Autophagy is an important component of the innate and adaptive immunity of macrophages, playing an important role in regulating intracellular protein and metabolic homeostasis. Appropriate autophagy can increase the defence ability of cells. However, under pathological conditions, the level of autophagy in cells can be changed, and changes in the degree of autophagy can lead to different consequences. Excessive autophagy can lead to cell death, while autophagy dysfunction has been found to be associated with a variety of diseases, including cancer and lung diseases (Shintani and Klionsky, 2004; Nakahira et al., 2011). LC3 is an important autophagy-related protein that occurs in cells in three forms: LC3 precursor, LC3-I, and LC3-II. When autophagy occurs, cytosolic LC3-I is translocated to the membrane of autophagosomes and converted into LC3-II, marking the formation of autophagosomes (Tanida et al., 2008). As the action centre of regulatory proteins and ubiquitinated protein aggregates, p62 is involved in the regulation of protein metabolism and multiple signal transduction pathways and is a ubiquitin-binding protector that isolates harmful proteins. Increased expression of p62 often suggests blockage of autophagic flux (Lamark et al., 2017). Many studies have shown that autophagy is involved in different types of lung injury, such as chronic obstructive pulmonary disease caused by cigarette smoke and ventilator-induced lung injury (Ryter et al., 2010; Gao et al., 2013). PM2.5 exposure can cause different types of cellular autophagy disorders, resulting in a variety of diseases. PM2.5 induces autophagy and apoptosis in human lung epithelial A549 cells through oxidative stress, leading to cell death (Deng et al., 2013). Wan et al. showed that PM2.5 can downregulate autophagy in macrophage RAW264.7 cells by activating the PI3K/Akt/mTOR signalling pathway, increase the concentration of inflammatory cytokines such as IL-6 and TNF-α in the supernatant, and accelerate atherosclerosis in mice (Wan et al., 2021). Our findings showed that concerted stimulation by PM2.5 and *P. aeruginosa* could lead to autophagy dysfunction in mouse alveolar macrophages, which could in turn reduce the defence ability of the cells and lead to severe inflammation. In addition, autophagy can inhibit or enhance apoptosis or induce cell death independent of apoptosis, depending on the cell type and the type and duration of stimulation (Delgado and Tesfaigzi, 2013). This study showed that the concerted action of PM2.5 and *P. aeruginosa* could inhibit autophagy and induce apoptosis. The specific mechanism between the two requires further study.

The mTOR signalling pathway is closely related to cell growth, proliferation, autophagy, apoptosis, and cytoskeletal rearrangement. mTOR, a serine/threonine protein kinase, is a central signalling regulatory molecule that integrates various intracellular and extracellular signals and regulates cell growth, metabolism, autophagy, and cytoskeletal rearrangement. Intracellular and extracellular signals (cytokines, growth factors, growth hormone, stress, etc.) can activate mTOR by stimulating the Akt signalling pathway (Zoncu et al., 2011). Akt, also known as protein kinase B, plays very important roles in regulating cell growth, proliferation, migration, and survival (Brown and Banerji, 2017). The mTOR signalling pathway can recruit and activate immune cells to secrete immune factors, thus playing an important role in the development and progression of inflammation (Felger, 2018). Some studies have shown that autophagy is regulated by the mTOR pathway, confirming that activation of the mTOR pathway can inhibit the occurrence of autophagy (Xu et al., 2020). Nicotine can inhibit autophagy and induce apoptosis of human alveolar epithelial cells by activating the mTOR pathway (He Li, 2021). Our study showed that both PM2.5 and *P. aeruginosa* can enhance the expression of Akt/mTOR proteins, and the enhancement effect is greatest when the two act synergistically. This finding suggests that PM2.5 and *P. aeruginosa* can destroy the cytoskeleton of alveolar macrophages and affect their phagocytic function by activating the mTOR pathway, thereby inhibiting autophagy and inducing apoptosis. Past studies have reported that there is crosstalk between the mTOR signalling pathway and the NF-κB pathway and that activated Akt can lead to activation of the NF-κB pathway and can enhance the activation of mTOR (Kim et al., 2007; Zoncu et al., 2011). As shown in our previous study, the lung tissues of mice exhibited greater pathological damage when costimulated by PM2.5 and *P. aeruginosa*, and this treatment increased the expression levels of IL-6, IL-8, and TNF-α through the NF-κB pathway. This finding demonstrated that PM2.5 in combination with *P. aeruginosa* can aggravate the inflammatory response by activating the NF-κB pathway (Li et al., 2020). The present study suggests that stimulation by PM2.5 in poultry houses in combination with *P. aeruginosa* can lead to abnormal expression of the Akt/mTOR signalling pathway and damage the function of alveolar macrophages. Moreover, activated Akt further activates the NF-κB pathway, causing the secretion of a large number of inflammatory cytokines, which aggravate the inflammatory response of the respiratory system, resulting in more severe lung injury.

## Conclusion

In summary, PM2.5 in poultry houses, together with *P. aeruginosa,* can activate the Akt/mTOR signalling pathway, disrupt the cytoskeleton of alveolar macrophages, affect phagocytosis, inhibit autophagy, induce apoptosis, and jointly regulate the inflammatory process through crosstalk with NF-κB, thereby further activating macrophages to secrete various inflammatory cytokines to produce more intense inflammatory responses. This outcome can explain the pathogenic mechanism by which, after PM2.5 in the poultry house damages alveolar macrophages, *P. aeruginosa*-induced secondary infection further aggravates the damage to alveolar macrophage function and exacerbates inflammatory responses. This finding provides a theoretical basis for preventing secondary infection by *P. aeruginosa*.

## Data Availability

The original contributions presented in the study are included in the article/supplementary materials, further inquiries can be directed to the corresponding author.
